# Severe Urosepsis Secondary to Xanthogranulomatous Pyelonephritis: A Case Report

**DOI:** 10.7759/cureus.15190

**Published:** 2021-05-23

**Authors:** Malak J Alzahrani, Abdulmalik A Alkhamis, Dunya Alfaraj

**Affiliations:** 1 Emergency Medicine, King Fahad Specialist Hospital, Dammam, SAU; 2 Urology, King Fahd Hospital of the University, Imam Abdulrahman Bin Faisal University, Dammam, SAU; 3 Emergency Medicine, King Fahd Hospital of the University, Imam Abdulrahman Bin Faisal University, Dammam, SAU

**Keywords:** xanthogranulomatous pyelonephritis, urosepsis, pyelonephritis, obstructive pyelonephritis, case report

## Abstract

Xanthogranulomatous pyelonephritis (XGP) is considered to be a rare variant of chronic pyelonephritis, which results in non-functioning kidneys in patients. The exact etiology of this disease is still unknown, and hence even its pathophysiology remains unclear. We present a case of a 27-year-old Saudi male patient who had been bed-bound with a known case of a congenital anomaly with severe kyphoscoliosis, bilateral lower limbs deformity with paraplegia, and a ventriculoperitoneal shunt since childhood. The patient was brought to the emergency department (ED) with right flank pain associated with fever and difficulty in breathing. The patient had a past medical history of recurrent urinary tract infection (UTI) with up to two incidences per year and renal stones. He had been recently discharged from the ICU of another hospital with sepsis due to UTI. An abdominal CT scan was performed, which showed a mass in the upper lobe of the right kidney measuring about 9 x 8 x 6 cm, suggestive of XGP. The final diagnosis was severe urosepsis secondary to right obstructive pyelonephritis. Patients with XGP usually present with nonspecific symptoms including back and abdominal pain, fever, UTI, and the condition is more common among middle-aged women. Ultimately, early detection and diagnosis, followed by prompt treatment with partial or total nephrectomy are associated witha good prognosis for patients with XGP.

## Introduction

Xanthogranulomatous pyelonephritis (XGP) is a rare variant of chronic pyelonephritis that leads to non-functioning kidneys in patients. The exact etiology of this disease is not well established and, consequently, its pathophysiology remains unclear. There are limited updated literature reviews discussing and reporting on this specific disease. Jha and Aeddula have reported that the incidence of XGP accounts for 0.6-1% of all cases with renal infections, and the disease can affect all age groups but is more common among women than men [1]. We present this case report to highlight how this rare disease can be aggressive and leads to severe complications that could seriously affect the patient's condition and could even be fatal if left untreated.

## Case presentation

**Figure 1:**
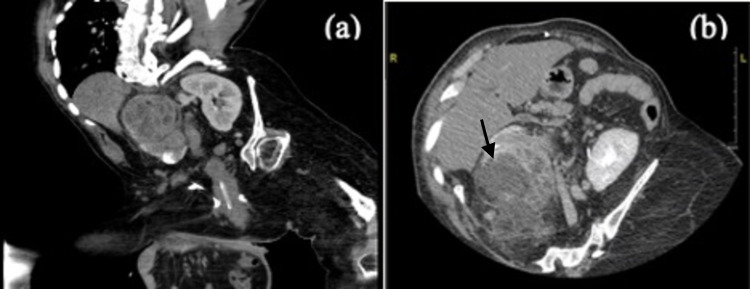
Patient’s abdominal CT scan (a) coronal view. (b) axial view. The images show a large mass in the upper lobe of the right kidney measuring about 9 x 8 x 6 cm, suggestive of xanthogranulomatous pyelonephritis CT: computed tomography

The patient was a 27-year-old Saudi male who had been bed-bound with a history of congenital anomaly of severe kyphoscoliosis, bilateral lower limbs deformity with paraplegia, and a ventriculoperitoneal shunt since early childhood. He was brought to the emergency department (ED) with right flank pain associated with fever and difficulty in breathing. The patient had no complaints of chest pain, palpitations, cough, rhinorrhea or nausea, and vomiting. He could not comment on symptomatic urinary issues due to his baseline paraplegic status. The patient had a past medical history of recurrent urinary tract infection (UTI) characterized by up to two incidences per year and renal stones. He had been discharged four days prior to this admission from a different hospital's ICU, with a diagnosis of sepsis. On physical examination, his vitals were as follows: temperature of 38 °C, heart rate of 165 beats per minute, respiratory rate of 28 breaths per minute, blood pressure of 144/95 mmHg, and oxygen saturation of 94% on room air. Cardiovascular examination revealed a normal S1+S2, no added sound, and intact peripheral pulses without peripheral edema. Chest examination showed bilateral decreased air entry with restricted chest movement due to kyphoscoliosis. Abdominal examination revealed a soft and lax abdomen with right upper quadrant tenderness. Lab investigations showed elevated WBC, metabolic acidosis, coagulopathy, elevated inflammatory markers (Table 1), and a positive urinalysis for UTI. His ECG demonstrated sinus tachycardia and pulmonary hypertension, and tricuspid regurgitation was found on his transthoracic echocardiogram. CT imaging of his chest and abdomen was performed with negative results for a pulmonary embolism but showed a mass on the right kidney suggestive of XGP (Figure 1).

**Table 1:** Patient’s lab investigations CBC: complete blood count; BUN: blood urea nitrogen; LDH: lactate dehydrogenase; PT: prothrombin time; PTT: partial thromboplastin time; CRP: C-reactive protein; ESR: erythrocyte sedimentation rate; FDP: fibrin degradation products

Investigations	Reference range	Patient results
CBC		
Total white blood cells, k/µL	4.0-11.0	33.3
Hemoglobin, g/dL	13.0-18.0	10.9
Platelets, k/ul	140-450	603
Biochemistry		
Creatinine, mg/dL	0.6-1.3	0.44
BUN, mg/dL	8.4-21	6
Ca+, mg/dL	8.4-10.2	8.1
K+, mEq/L	3.5-5.1	4.4
CL-, mEq/L	98-107	94
LDH, units/L	81-234	344
Arterial blood gases		
pH	7.35-7.45	7.16
pO_2, _mmHg	83-108	148
pCO_2, _mmHg	35-45	50.3
HCO_3, _mmol/L	22-26	16.3
Coagulation profile		
PT, seconds	13-16.5	18.6
PTT, seconds	25.6-42.3	46.6
Lactic acid, mmol/L	0.5-2.2	4.31
CRP, mg/dL	0.10-0.5	30.89
Procalcitonin, ng/mL	<0.1	445.81
ESR, mm/hour	0-20	91
Fibrinogen	200-400	553
FDP, ug/ml	<5.00	14.7
Plasma D-dimer, ug/ml	<0.5	3.35

The patient was started on 1000 mL of 0.9% normal saline IV, paracetamol 1 g IV, and ceftriaxone 1000 mg IV in the ED. He was referred to the urology department as a case of severe urosepsis secondary to right obstructive pyelonephritis for nephrectomy after stabilization, and he received 2 g of meropenem in the ED. Both the blood cultures and urine culture collected from his foley catheter showed no growth after 24 hours, and the coronavirus swab returned negative. The patient was transferred to the surgical ICU due to oxygen desaturation requiring 5 liters of oxygen via nasal cannula. He then became hemodynamically unstable within hours of admission and subsequently passed away.

## Discussion

According to the previously published literature, bilateral XGP is an extremely rare condition, with middle-aged women more frequently affected than men. Additionally, up to 80.5% of the affected kidneys in these patients have been found to be nonfunctioning [2]. A study conducted by Leoni et al. involving 10 patients with XGP found that 80% of patients presented with nonspecific symptoms, including back and abdominal pain, weight loss, conjunctival pallor, renal lithiasis, fever, and palpable abdominal mass. On the other hand, 60% presented with a history of UTI [3]. Moreover, Nawaz et al. found that 93.6% of patients presented with fever and flank pain, and 53.9% of patients presented with pyuria [4]. In our case, the patient had multiple previous incidences of recurrent simple UTIs, flank pain, and fever, and he had been usually managed with an oral antibiotic course, without the need for hospitalization.

In the case series by Dwivedi et al., 100% of XGP patients showed decreased hemoglobin levels (<10 g/dL) [5]. Another laboratory finding that is typical in these patients is an elevated creatinine level. However, the creatinine level can be normal as long as the other kidney is functioning normally. Additionally, leukocytosis and pyuria can also be found in the urine of these patients [6]. Similarly, our patient's results showed many typical findings of XGP, including a decreased hemoglobin level (although higher than 10 g/dl), normal creatinine, and urinalysis with an elevated WBC. Nicola and Menias have found that the most common inciting organisms in XGP patients are *Escherichia coli (E. coli) *and *Proteus mirabilis*, which are organisms often associated with infected struvite stones. Patients with the extrarenal extension of XGP often present with inflammatory changes and abscess, usually located in the posterior flank, psoas muscle, and adjacent visceral organs (liver and spleen) [7].

The ultrasound features of XGP are nonspecific, including focal or diffuse enlargement of the kidneys, and hence contrast-enhanced CT is considered the modality of choice for its evaluation. Complete nephrectomy is the recommended standard of care for the diffuse form and partial nephrectomy is suggested for the focal disease [8]. Karkar has reported a case of XGP with symptoms similar to our patient, including nonspecific left groin pain and increased micturition, which ultimately led to Gram-negative sepsis. Our final diagnosis for our patient was left XGP with left ureteric stone, Gram-negative urosepsis complicated by acute kidney injury, nephrogenic hepatic dysfunction, disseminated intravascular coagulation (DIC), and Methicillin-resistant *Staphylococcus aureus* (MRSA) infection of the retroperitoneal space [9]. Although XGP has a good prognosis if it is unilateral, delayed presentation and management could be fatal due to the development of complications related to sepsis and multisystem organ failure.

XGP can be classified according to its extension into three stages (Malek and Elder classification) - stage I: disease confined to renal parenchyma only; stage II: disease involves perinephric fat along with renal parenchyma; and stage III: disease extending into retroperitoneum with involvement of adjacent structure. Thus, the stage of the disease can affect the outcome [10].

Urosepsis and obstructed pyelonephritis should still be considered as a serious threat, and these have a high mortality rate exceeding 40%. Rapid initiation of appropriate antimicrobial therapy and percutaneous nephrostomy tube placement are crucial for decreasing the risk of mortality [11]. In our case, delayed presentation to the hospital and consequently delayed management poorly affected the patient's outcome, and it contributed to major complications in care.

## Conclusions

Although XGP is a rare entity, it is a chronic condition that could eventually lead to the destruction and replacement of the renal parenchyma, and it could also invade other organs. It should be considered in the differential diagnoses for patients with pelvic stones and enlarged kidneys, even in the absence of fever, bodyweight loss, and recurrent UTI. Moreover, early detection and diagnosis, followed by proper treatment including either total or partial nephrectomy, are associated with a good prognosis and a favorable outcome for the patients.
